# A phase II study of paclitaxel and capecitabine as a first-line combination chemotherapy for advanced gastric cancer

**DOI:** 10.1038/sj.bjc.6604186

**Published:** 2008-01-22

**Authors:** H J Kang, H M Chang, T W Kim, M-H Ryu, H-J Sohn, J H Yook, S T Oh, B S Kim, J-S Lee, Y-K Kang

**Affiliations:** 1 Division of Oncology, Department of Medicine, University of Ulsan College of Medicine, Asan Medical Center, Seoul, Korea; 2 Department of Surgery, University of Ulsan College of Medicine, Asan Medical Center, Seoul, Korea

**Keywords:** paclitaxel, capecitabine, advanced gastric cancer

## Abstract

Paclitaxel and capecitabine, which have distinct mechanisms of action and toxicity profiles, have each shown high activity as single agents in gastric cancer. Synergistic interaction between these two drugs was suggested by taxane-induced upregulation of thymidine phosphorylase. We, therefore, evaluated the antitumour activity and toxicities of paclitaxel and capecitabine as first-line therapy in patients with advanced gastric cancer (AGC). Patients with histologically confirmed unresectable or metastatic AGC were treated with capecitabine 825 mg m^−2^ p.o. twice daily on days 1–14 and paclitaxel 175 mg m^−2^ i.v. on day 1 every 3 weeks until disease progression or unacceptable toxicities. Between June 2002 and May 2004, 45 patients, of median age 57 years (range=38–73 years), were treated with the combination of capecitabine and paclitaxel. After a median 6 cycles (range=1–9 cycles) of chemotherapy, 43 were evaluable for toxicity and response. A total of 2 patients showed complete response and 20 showed partial response making the overall response rate 48.9% (95% CI=30.3–63.5%). After a median follow-up of 42.2 months (range=31.2–54.3 months), median time to progression was 5.6 months (95% CI=3.9–7.2 months) and median overall survival was 11.3 months (95% CI=8.1–14.4 months). Grade 3 or 4 adverse events include neutropaenia (46.5% of patients), hand–foot syndrome (9.3%), arthralgia (9.3%), and asthenia (4.7%). There was no neutropaenic fever or treatment-related deaths. Paclitaxel and capecitabine combination chemotherapy was active and highly tolerable as a first-line therapy for AGC.

Although the overall incidence of gastric cancer has steadily declined in many Western countries during the last few decades (Jemal *et al*, 2002), it is still the most common tumour in Korea (Bae *et al*, 2002). Gastric cancer is often diagnosed at a very advanced stage, with approximately half of all patients presenting with unresectable, locally advanced, or metastatic disease. For patients with advanced gastric cancer (AGC), the median survival is only 6–10 months, and 5-year survival rates are <10%.

Four randomised studies comparing best-supportive care with best-supportive care plus chemotherapy for AGC have shown that chemotherapy can improve survival and quality of life (Murad *et al*, 1993; Pyrhonen *et al*, 1995; Schipper and Wagener, 1996; Glimelius *et al*, 1997). The combination of 5-FU plus cisplatin (FP) resulted in improved response rates compared with 5-FU, doxorubicin and mitomycin (FAM) or 5-FU single-agent therapy (Kim *et al*, 1993), and showed a trend towards improved response rates when compared with 5-FU, doxorubicin and methotrexate (FAMTX) or etoposide, leucovorin and bolus 5-FU (ELF) (Vanhoefer *et al*, 2000). The combination of epirubicin, cisplatin, and infusional 5-FU (ECF) led to longer survival than the combination of 5-FU, doxorubicin, and high-dose methotrexate (FAMTX) (median overall survival (OS) 8.9 months *vs* 5.8 months) (Webb *et al*, 1997). Thereafter, these two FP-based chemotherapy regimens (FP and ECF) became a standard reference regimen in first-line treatment for AGC. Nonetheless, the treatment outcomes with these regimens were not satisfactory either in efficacy or safety, such as inconvenience and complication associated with portable pump for administration of infusional 5-FU, and nausea/vomiting and neurotoxicity related with cisplatin. So, development of more effective or better tolerable chemotherapy regimens have been an urgent task in AGC.

Paclitaxel (Taxol®; Bristol–Meyers Squibb Company, Princeton, NJ, USA), the prototype taxane compound that interferes with tubulin assembly (Rowinsky *et al*, 1990), has been studied extensively in patients with previously treated or untreated gastric cancer. As a single agent, paclitaxel induced responses in 11–17% of previously untreated patients (Ajani *et al*, 1998; Garcia *et al*, 2001), and activity was also seen in previously treated patients (Cascinu *et al*, 1998; Yamada *et al*, 2001). The *in vitro* cytotoxic effects of paclitaxel plus 5-FU were found to depend on the schedule used, in that application of paclitaxel before 5-FU enhanced cytotoxicity, whereas the application of paclitaxel after 5-FU resulted in a less than additive cytotoxic effect (Kano *et al*, 1996). In patients with AGC, the combination of these two drugs had response rates between 13 and 65.5%, with a rather low-toxicity profile (Cascinu *et al*, 1997; Murad *et al*, 1999).

Capecitabine (*N*4-pentoxycarbonyl-5′-deoxy-5-fluorocytidine; Xeloda®; Hoffmann-La Roche Ltd, Basel, Switzerland) is a 5-FU prodrug developed to reduce the toxicity and enhance the intratumour concentrations of 5-FU. Capecitabine is absorbed as an intact molecule from the small bowel mucosa and converted sequentially to 5-FU in a multistep enzymatic process (Bollag and Hartmann, 1980; Hartmann and Bollag, 1980). In the first step, capecitabine is metabolised by hepatic carboxyl esterase to 5′-deoxy-5-fluorocytidine (5′-DFCR). This intermediate is metabolised by cytidine deaminase to doxifluridine (5′-DFUR) in hepatic and extrahepatic tissues, including malignant tumours. Finally, 5′-DFUR is converted to 5-FU by the pyrimidine nucleoside phosphorylase thymidine phosphorylase (dThdPase), a potent tumour-associated angiogenesis factor preferentially expressed in malignant cells (Ishikawa *et al*, 1998a). In preclinical xenograft models, capecitabine was highly active against several tumours, including breast, colorectal, gastric, and cervical tumours (Ishikawa *et al*, 1998b; Ishitsuka, 2000), and against both 5-FU-sensitive and 5-FU-resistant tumours (Cao *et al*, 1997). Intermittent capecitabine (1250 mg m^−2^ daily dose for 14 days, followed by a 7-day rest period) was shown to be active as a single agent in previously untreated AGC patients, with a response rate of 28.2% in 39 patients (Hong *et al*, 2004). Moreover, the combination of capecitabine with other drugs, such as cisplatin, oxaliplatin, epirubicin, and docetaxel, had an objective response rate of 40–68% as first-line treatment in patients with AGC (Kim *et al*, 2002; Kang *et al*, 2004; Park *et al*, 2004, 2006; Cho *et al*, 2005).

In human colon cancer xenograft model, thymidine phosphorylase is upregulated and synergy between paclitaxel and capecitabine has been observed (Sawada *et al*, 1998). The activity of capecitabine in patients with breast cancer refractory to paclitaxel and anthracyclines suggests that the combination of capecitabine and paclitaxel may be effective in treating patients with advanced breast cancer (Blum *et al*, 1999). Doses recommended are capecitabine 1650 mg m^−2^ per day orally for 14 days and paclitaxel 175 mg m^−2^ i.v. every 3 weeks (Villalona-Calero *et al*, 2001).

We, therefore, performed a phase II study to evaluate the antitumour activity and toxicities of the combination of paclitaxel and capecitabine as first-line therapy in patients with AGC.

## PATIENTS AND METHODS

### Patient selection

Patients with histologically confirmed AGC, with at least one measurable lesion of longest diameter ⩾2 cm, were considered eligible for this study. In addition, patients 18–75 years old with ECOG performance status of 0–2 and adequate liver, renal, and bone marrow functions were eligible. Prior chemotherapy for advanced disease was not permitted, but adjuvant chemotherapy was allowed, providing it was completed at least 6 months before the start of study treatment. Patients were excluded if they had been previously exposed to taxane although fluoropyrimidine was allowed as adjuvant therapy. Patients with unresolved bowel obstruction or malabsorption syndrome were excluded. The protocol was approved by the Institutional Review Board of the Asan Medical Center, and all patients provided written informed consent before enrollment.

### Treatment schedule

Treatment consisted of i.v. paclitaxel 175 mg m^−2^ (diluted in 500 ml of 0.9% sodium chloride solution) for 3 h on day 1, followed by oral capecitabine 825 mg m^−2^ twice daily from the evening of day 1 to the morning of day 15, followed by a 7-day treatment-free interval, in each 3-week cycle (Villalona-Calero *et al*, 2001). Patients received standard i.v. hypersensitivity prophylaxis, including dexamethasone 20 mg, diphenhydramine 50 mg, and ranitidine 50 mg, 30 min before administration of paclitaxel.

Patients with response or stable disease received a maximum of 9 cycles of chemotherapy, or until disease progression, unacceptable toxicity, or refusal by the patient. Patients withdrawing from the study due to adverse effects of study drugs could continue on monotherapy.

### Dose modification for adverse events

Toxicity was evaluated before each treatment cycle according to the National Cancer Institute Common Toxicity Criteria (NCI CTC), version 2.0. To begin the next treatment cycle, each patient was required to have a platelet count ⩾100 × 10^9^ l^−1^, an absolute neutrophil count ⩾1.5 × 10^9^ l^−1^ and resolution or improvement of clinically significant non-haematological adverse events, except alopecia, to grade 1 or 0. A treatment delay of up to 1 week was permitted without dose reduction.

Treatment was continued at the same dose, without interruption or dose reduction, in patients experiencing grade 1 or other toxicities considered unlikely to become serious or life threatening (e.g., alopecia). For all other treatment-related adverse events of grade 2 or higher (except grade 3 peripheral neuropathy or neutropaenia, as described below), a dose modification scheme was implemented. Dose reduction was not required following the first appearance of any grade 2 toxicity, although treatment was interrupted/delayed until the toxicity had resolved to grades 0–1 and symptomatic treatment was initiated when possible. Treatment with both agents was interrupted/delayed and the dose of both agents was reduced by 25% in patients who experienced a second occurrence of any grade 2 toxicity or at the first occurrence of a grade 3 toxicity. If patients experienced a third occurrence of any grade 2 toxicity or a second occurrence of any grade 3 toxicity, treatment was interrupted/delayed until the toxicity resolved to grades 0–1 and the dose of both agents was reduced by 50%. Treatment with both agents was discontinued if, despite dose reduction, any grade 2 toxicity occurred for a fourth time or any grade 3 toxicity for a third time. Treatment was also discontinued if patients experienced a grade 4 non-haematological toxicity.

Paclitaxel was discontinued and capecitabine treatment was modified according to the scheme outlined above in patients experiencing grade 3 peripheral neuropathy. Paclitaxel dose was permanently reduced by 25% for patients who developed grade 4 neutropaenia for more than 5 days, or grade 3 or 4 neutropaenia associated with a temperature of ⩾38°C. Paclitaxel was discontinued if patients receiving the reduced dose experienced grade 4 or febrile neutropaenia. Patients with grade 4 thrombocytopaenia were retreated with a 25% dose reduction after recovery. As capecitabine was not expected to worsen or prolong neutropaenic episodes, treatment with this agent could be continued during episodes of grade 3–4 neutropaenia. However, capecitabine was interrupted if any other grade 2 toxicity developed during the neutropaenic episodes.

### Assessment of compliance and dose intensity

Compliance to capecitabine treatment was monitored by questioning patients and counting their remaining pills at each outpatient visit. The ratio of the actual administered dose to the scheduled dose was calculated. Dose intensity was defined as the total amount of drug given (mg m^−2^) divided by the number of weeks.

### Pretreatment, follow-up studies and response evaluation

Prestudy screening assessments, completed within the 3 weeks preceding treatment, included a full medical history, vital signs and physical measurements, haematological and blood chemistry tests, electrocardiogram, chest X-ray, and computed tomography (CT) scans.

Complete blood counts with differential counts were performed every week to assess haematological toxicities, and physical examinations and biochemical tests were performed before each chemotherapy cycle. Response evaluation was performed by CT scan every 2 cycles until disease progression or withdrawal from study medication. Tumour response was classified on the basis of the response evaluation criteria in solid tumours guidelines (Therasse *et al*, 2000), with responses confirmed as lasting longer than 4 weeks.

### Statistical analysis

All enrolled patients were included in the intention-to-treat (ITT) analysis of efficacy. This trial was designed using Simon’s two-stage phase II designs (Simon, 1987). Assuming a target level of interest, *p*_1_=0.5, and a lower activity level, *p*_0_=0.3, we planned to enroll 19 patients initially. If seven or more responses were observed, the trial would be continued. Accrual would be planned to a total of 44 patients assuming a 10% dropout rate due to protocol non-compliance. This design provides a probability ⩽0.05 of accepting drugs worse than *p*_0_ and a probability ⩽0.20 of rejecting drugs better than *p*_1_.

Time to progression (TTP), survival and duration of response were estimated as secondary end points by the Kaplan–Meier method. The duration of response was defined as the interval from the onset of complete response (CR) or partial response (PR) until first appearance of evidence of progression. Time to progression was calculated from the date of entry into the study until the date of progression, and OS was measured from the date of entry to the date of last follow-up or death.

## RESULTS

### Patient characteristics

A total of 45 patients were enrolled from June 2002 to May 2004, and their baseline characteristics are shown in Table 1. Their median age was 57 years, and 42 of 45 patients (93.3%) had a good performance status (ECOG 0 or 1). Ten patients had recurrent disease after previous curative gastrectomy and nine had previous adjuvant chemotherapy (three FAM, five doxifluridine or 5-FU plus cisplatin, and one doxifluridine plus mitomycin-C). The median disease-free interval of relapsed patients was 33.0 months (range=16.4–55.6 months). Twenty-six patients (57.8%) had multiple metastases involving two or more organs, with the abdominal lymph nodes and liver being the most common sites of metastases.

### Efficacy and survival

A total of 43 patients were assessable for response (Table 2). Of the two patients not assessable, one was lost to follow-up after the first cycle of treatment, and the other died after the first cycle of unknown cause, although brain metastasis was suspected. Of the 43 assessable patients, 2 achieved CR and 20 achieved PR, giving an overall response rate of 48.9% (95% CI=30.3–63.5%) in the ITT population.

**Figure 1:**
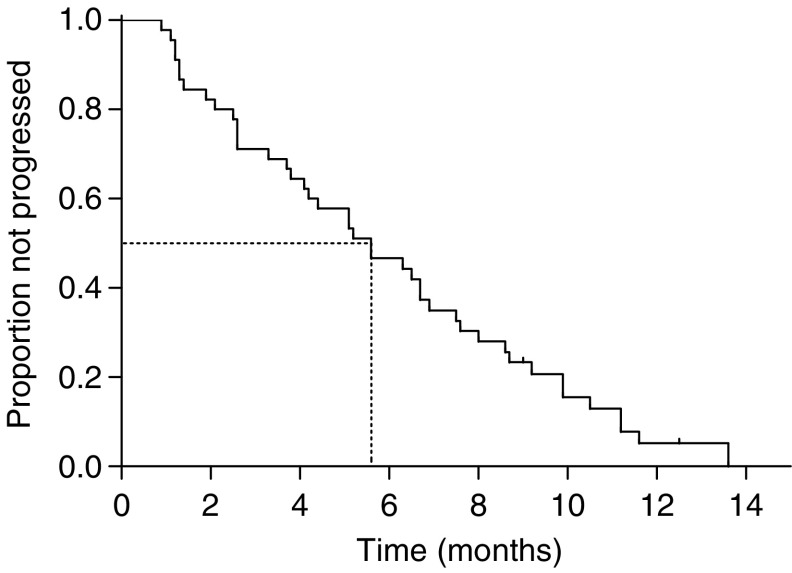
Time to progression for all patients.

**Figure 2:**
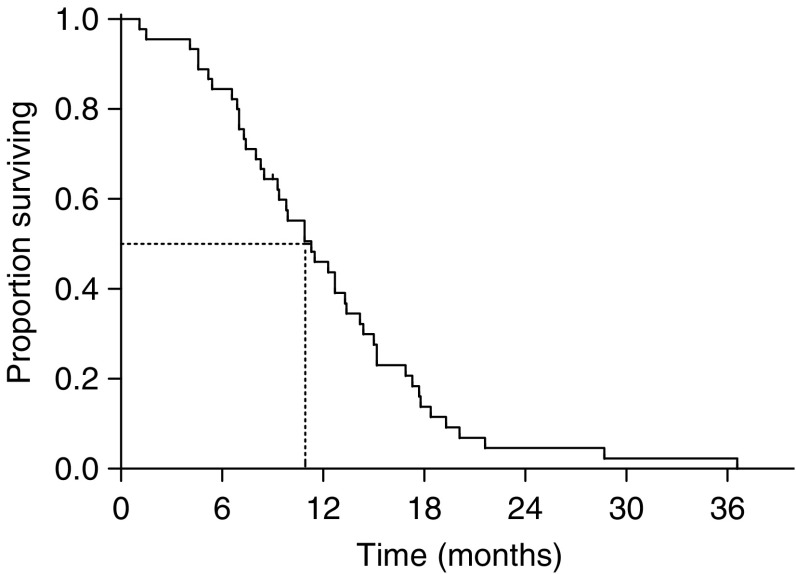
Overall survival for all patients.

There was no difference in overall response rate between patients who were pretreated and who were not with adjuvant chemotherapy (33.3 *vs* 52.8%, *P*=0.459 by Fisher’s exact test). The median duration of response in the 22 responding patients was 6.1 months (range=2.9–12.4 months). The median follow-up period was 42.2 months (range=31.2–54.3 months). The median TTP for all patients was 5.6 months (95% CI=3.9–7.2 months) (Figure 1), and the median OS was 11.3 months (95% CI=8.1–14.4 months) (Figure 2), with a 1-year survival rate of 46.0% (95% CI=31.4–60.6%).

### Poststudy treatment

After disease progression, 28 patients (62.2%). received second-line chemotherapy, most commonly with irinotecan (21 patients; 75%) in combination with cisplatin/mitomycin-C or 5-FU/LV. Two patients (7.1%) achieved PR and 5 (17.9%) had stable disease in response to second-line chemotherapy, the median TTP was 1.5 months (95% CI=1.3–1.8 months). Three patients (6.7%) received palliative radiotherapy, and one patient underwent total gastrectomy due to cancer bleeding.

### Adverse events

A total of 248 treatment cycles (median=6; range=1–9 cycles) were administered, with 246 cycles and 43 patients assessable for safety. The frequencies of haematological and non-haematological adverse events are shown in Table 3 and 4, respectively. The most common haematological adverse event was neutropaenia, which occurred at grade 3 or 4 intensity in 20 patients (46.5%) and during 51 cycles (20.7%). No patient experienced febrile neutropaenia. There were no treatment-related deaths.

Non-haematological adverse events included asthenia (100%), alopecia (98%), neuropathy (93%), arthralgia (86%), nausea (74%), hand–foot syndrome (72%), myalgia (67%), and constipation (65%), but grade 3 or 4 non-haematological adverse events were rare. Treatment was delayed or the dose was reduced during 34 cycles. Treatment was delayed during 5 cycles (2.0%) and in 5 patients (11.6%), due to neutropaenia (2 cycles), elevated transaminase (2 cycles), and hand–foot syndrome (1 cycle). Paclitaxel dose was reduced during 14 cycles (5.6%) in 4 patients (9.3%), due to myalgia (8 cycles), arthralgia (4 cycles), neutropaenia (1 cycle) and neuropathy (1 cycle). The dose of capecitabine was reduced during 25 cycles (10.1%) in 6 patients (14%), due to hand–foot syndrome (17 cycles), and myalgia (8 cycles).

Ten patients (22.2%) withdrew from study treatment because of financial problems or paclitaxel-induced neurotoxicity. These 10 patients received a median 3.5 cycles (range=1–9 cycles) of capecitabine monotherapy.

**Figure 3:**
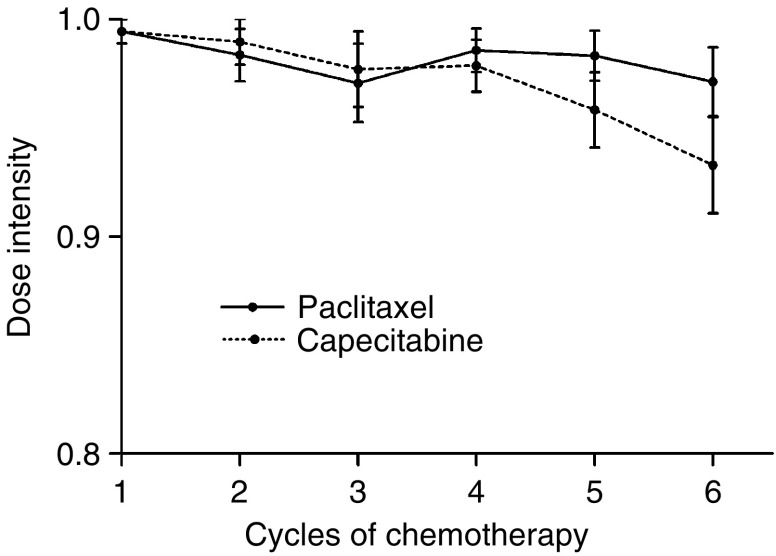
Dose intensity of paclitaxel and capecitabine during cycles 1–6.

Over all treatment cycles, the mean dose intensity of paclitaxel was 57.2 mg m^−2^ per week (range=23.3–58.3 mg m^−2^ per week) and that of capecitabine was 7277 mg m^−2^ per week (range=3080–7700 mg m^−2^ per week), corresponding to 98.0 and 94.5%, respectively of the planned dose intensities. The actual dose intensity of both drugs was maintained at over 95% during the first 5 chemotherapy cycles (Figure 3). There was 97.5% compliance with capecitabine treatment during the first 6 cycles.

## DISCUSSION

The results presented here suggest that the combination of paclitaxel and capecitabine is effective and well tolerated as a first-line regimen in patients with AGC. This combination regimen demonstrated promising efficacy, with a tumour response rate of 48.9%, a median TTP of 5.6 months, and a median OS of 11.3 months. These results are comparable to other paclitaxel-based combination regimens in patients with previously untreated AGC. For example, in 29 evaluable patients treated with paclitaxel 175 mg m^−2^ on day 1 followed by 5-fluorouracil 1500 mg m^−2^ on day 2 every 3 weeks, the response rate 65.5% and the median OS was 12 months (Murad *et al*, 1999), and the combination of paclitaxel with a 24-h continuous infusion of high-dose 5-FU/folinic acid and cisplatin in 45 evaluable patients resulted in a 51% response rate, a median TTP of 9 months, and a median OS of 14 months (Kollmannsberger *et al*, 2000).

The results of this combination of paclitaxel and capecitabine (TX) seem to be also comparable in efficacy and even better in safety as compared to the current standard platinum-containing first-line regimens. In comparison with capecitabine plus cisplatin (XP) or 5-FU plus cisplatin (FP) combination reported in a recent phase III trial (Kang *et al*, 2006), this TX regimen was comparable in terms of RR (48.9 *vs* 46/32%), TTP (5.6 months *vs* 5.6/5.0 months), and OS (11.3 months *vs* 10.5/9.3 months), and even better in terms of risk of severe nausea/vomiting (0 *vs* 6/8%). Similarly, in comparison to ECF of REAL-2 trial (Cunningham *et al*, 2006), in addition to comparable efficacy, this TX regimen seems to be very safe, especially in terms of infection risk. There was no febrile neutropaenia and treatment-related death with TX regimen, while there was 6.7–9.3% of febrile neutropaenia with ECF, ECX, EOF, and EOX regimen in REAL-2 trial.

Two trials of paclitaxel/capecitabine combination in patients with metastatic breast cancer (MBC) showed similar toxicities with asthenia, alopecia, and hand–foot syndrome being common (Batista *et al*, 2004; Gradishar *et al*, 2004). Although we found that the incidence of grade 3 or 4 hand–foot syndrome and neuropathy were similar in MBC and AGC patients, grade 3 or 4 neutropaenia was lower in MBC (12 and 15%) than in AGC (46.5%) patients. This may have resulted from an underestimation in MBC patients due to CBC evaluations every 3 weeks.

We found that the rate of nail toxicity (all grades) was 37.2% and the rate of grade 3 hand–foot syndrome was 9.3%. In comparison, the combination of docetaxel 75 mg m^−2^ i.v. on day 1 and capecitabine 1250 mg m^−2^ p.o. twice daily on days 1–14 every 3 weeks resulted in rates of oncolysis (all grades) of 81% and of grade 3 hand–foot syndrome of 50% (Park *et al*, 2004). These differences may have resulted from the lower dose of capecitabine in our regimen and from the use of docetaxel. Although it is difficult to compare the incidence of neutropaenia, we observed no incidence of febrile neutropaenia, whereas the previous trial reported febrile neutropaenia in three patients (7%). These findings suggest that the combination of paclitaxel and capecitabine may have a superior safety and tolerability profile than the combination of docetaxel and capecitabine.

In previously untreated patients, single-agent docetaxel has demonstrated response rates of 18, 20, and 24% when given at 100 mg m^−2^ (Sulkes *et al*, 1994; Einzig *et al*, 1996; Mavroudis *et al*, 2000), and 18% when given at 75 mg m^−2^ (Bang *et al*, 2002). Final results of a randomised phase III trial in chemotherapy-naive patients with locally advanced or metastatic gastric cancer showed that DCF (docetaxel/cisplatin/5-FU) was superior to CF (cisplatin/5-FU) in response rate (37 *vs* 25%), TTP (5.6 months *vs* 3.7 months) and OS (9.2 months *vs* 8.6 months) (Van Cutsem *et al*, 2006). However, the haematological toxicity in the DCF arm was significant, with grade 3 or 4 neutropaenia and febrile neutropaenia rates of 82.3 and 30.0%, respectively, suggesting that the DCF regimen has questionable clinical relevance as a standard regimen in patients with AGC.

The lower toxicities, very good compliance and higher dose intensities demonstrated in the present study suggests that dose escalation of capecitabine should be considered in AGC patients. However, a recent retrospective analysis of the impact of dose reduction in MBC patients treated with capecitabine suggests that the dose of capecitabine can be reduced to minimise toxicity without compromising efficacy (O’Shaughnessy and Blum, 2000).

In summary, we have shown that the combination of paclitaxel and capecitabine is active and highly tolerable as first-line chemotherapy for AGC. Response rates, TTP, and OS compare favourably with previous studies of paclitaxel/5-FU. Replacing infusional 5-FU with oral capecitabine improved convenience and allowed treatment in an outpatient setting.

## Figures and Tables

**Table 1:** Patient characteristics (*n*=45)

**Characteristic**	**No.**	**%**
*Age (years)*		
Median	57	
Range	20–73	
		
*Sex*		
Male	32	71.1
Female	13	28.9
		
*ECOG performance status*		
0	7	15.6
1	35	77.8
2	3	6.7
		
*Extent of disease*		
Relapsed	10	22.2
Initially metastatic	33	73.3
Initially locally advanced	2	4.4
		
*Prior adjuvant chemotherapy*		
Yes	9	20
5-FU+doxorubicin+mitomycin-C	−3	
5-FU or doxifluridine+cisplatin	−5	
Doxifluridine+mitomycin-C	−1	
No	36	80
		
*Metastatic sites*		
Abdominal lymph node	33	73.3
Liver	20	44.4
Peritoneum	12	26.7
Lung	7	15.6
Cervical lymph node	7	15.6
Ovary	3	6.7
		
*No. of metastases*		
1	19	42.2
2	15	33.3
⩾3	11	24.5

American Joint Committee on Cancer, 2002.

**Table 2:** Antitumour activity

	**All patients^a^ (*n*=45)**		**History of previous adjuvant chemotherapy**			
			**Yes (*n*=9)**		**No (*n*=36)**	
**Response**	**No.**	**(%)**	**No.**	**(%)**	**No.**	**(%)**
CR	2	4.4	1	11.1	1	2.8
PR	20	44.5	2	22.2	18	50.0
SD	15	33.3	6	66.7	9	25.0
PD	6	13.3	0	0	6	16.7
NA^b^	2	4.4	0	0	2	5.6

a Intention-to-treat analysis.

b Not assessable.

**Table 3:** Haematological toxicity (by patients and cycles)

	**Grade (% of patients, *n*=43)^a^**				**Grade (% of cycles, *n*=246)^a^**			
	**1**	**2**	**3**	**4**	**1**	**2**	**3**	**4**
Anaemia	46.5	39.5	7.0	0	53.7	27.6	2.0	0
Leukopaenia	32.6	37.2	2.3	0	29.3	17.9	0.4	0
Neutropaenia^b^	9.3	20.9	39.5	7.0	12.2	19.5	19.1	1.6
Thrombocytopaenia	7.0	0	0	0	3.3	0	0	0

a NCI CTC version 2.0.

b No febrile neutropaenia or treatment-related death.

**Table 4:** Non-haematological toxicity (by patients and cycles)

	**Grade (% of patients, *n*=43)^a^**				**Grade (% of cycles, *n*=246)^a^**			
	**1**	**2**	**3**	**4**	**1**	**2**	**3**	**4**
Nausea	53.5	20.9	0	0	30.1	6.1	0	0
Vomiting	20.9	16.3	0	0	8.9	3.7	0	0
Stomatitis	16.3	11.6	0	0	6.9	2.4	0	0
Diarrhoea	44.2	0	0	0	13.8	0	0	0
Constipation	48.8	14.0	2.3	0	20.7	3.7	0.4	0
Asthenia	58.1	37.2	4.7	0	72.4	11.8	0.8	0
Alopecia	0	97.7	—	—	4.1	82.1	—	—
Neuropathy	48.8	41.9	2.3	0	53.7	18.7	1.2	0
Hand–foot syndrome	46.5	16.3	9.3	0	31.7	10.2	2.8	0
Nail toxicity	23.3	11.6	2.3	0	17.1	8.9	0.4	0
Myalgia	48.8	16.3	2.3	0	32.9	7.7	0.4	0
Arthralgia	53.5	23.3	9.3	0	32.5	11.0	1.6	0
Elevated transaminase	14.0	2.3	0	0	4.9	0.8	0	0
Hyperbilirubinaemia	14.0	4.7	0	0	7.3	1.6	0	0

a NCI CTC version 2.0.
